# Desire to Be Underweight: Exploratory Study on a Weight Loss App Community and User Perceptions of the Impact on Disordered Eating Behaviors

**DOI:** 10.2196/mhealth.6683

**Published:** 2017-10-12

**Authors:** Elizabeth Victoria Eikey, Madhu C Reddy, Kayla M Booth, Lynette Kvasny, Johnna L Blair, Victor Li, Erika S Poole

**Affiliations:** ^1^ Donald Bren School of Information and Computer Sciences Department of Informatics University of California, Irvine Irvine, CA United States; ^2^ Department of Communication Studies Northwestern University Evanston, IL United States; ^3^ School of Computing and Information University of Pittsburgh Pittsburgh, PA United States; ^4^ College of Information Sciences and Technology The Pennsylvania State University University Park, PA United States; ^5^ University of Washington Seattle, WA United States; ^6^ Healthwise Boise, ID United States

**Keywords:** mobile applications, health apps, feeding and eating disorder, disordered eating behaviors, desire to be underweight, body mass index, weight loss, smartphone, mHealth, online community, forum, profile, posts, human computer interaction

## Abstract

**Background:**

Mobile health (mHealth) apps for weight loss (weight loss apps) can be useful diet and exercise tools for individuals in need of losing weight. Most studies view weight loss app users as these types of individuals, but not all users have the same needs. In fact, users with disordered eating behaviors who desire to be underweight are also utilizing weight loss apps; however, few studies give a sense of the prevalence of these users in weight loss app communities and their perceptions of weight loss apps in relation to disordered eating behaviors.

**Objective:**

The aim of this study was to provide an analysis of users’ body mass indices (BMIs) in a weight loss app community and examples of how users with underweight BMI goals perceive the impact of the app on disordered eating behaviors.

**Methods:**

We focused on two aspects of a weight loss app (DropPounds): profile data and forum posts, and we moved from a broader picture of the community to a narrower focus on users’ perceptions. We analyzed profile data to better understand the goal BMIs of all users, highlighting the prevalence of users with underweight BMI goals. Then we explored how users with a desire to be underweight discussed the weight loss app’s impact on disordered eating behaviors.

**Results:**

We found three main results: (1) no user (regardless of start BMI) starts with a weight gain goal, and most users want to lose weight; (2) 6.78% (1261/18,601) of the community want to be underweight, and most identify as female; (3) users with underweight BMI goals tend to view the app as positive, especially for reducing bingeing; however, some acknowledge its role in exacerbating disordered eating behaviors.

**Conclusions:**

These findings are important for our understanding of the different types of users who utilize weight loss apps, the perceptions of weight loss apps related to disordered eating, and how weight loss apps may impact users with a desire to be underweight. Whereas these users had underweight goals, they often view the app as helpful in reducing disordered eating behaviors, which led to additional questions. Therefore, future research is needed.

## Introduction

### Motivation

While it is estimated that 30 million people in the United States have an eating disorder, many more have disordered eating behaviors, especially young women [1-3]. Research has shown that people with eating disorders and disordered eating behaviors utilize various forms of technology, including forums, websites, blogs, and social media, to support their recovery process or maintain the symptoms of their disorder [4-6]. However, only a few studies have been conducted on the use of mobile health (mHealth) apps for weight loss (weight loss apps) by users with eating disorders and disordered eating behaviors, despite their popularity [7].

Although many researchers have studied weight loss apps [8-13], few have studied weight loss apps in relation to disordered eating behaviors [14,15]. Most research on weight loss apps focuses on the use and design of these apps to encourage overall wellness, healthy diet, weight loss, and physical activity. In terms of eating disorders and disordered eating, researchers have begun to consider the use of weight loss apps [14,15], but studies to date do not highlight the prevalence of users with disordered eating behaviors within weight loss app communities.

Many studies on the role of technology for disordered eating behaviors focus on content that is in support of eating disorders or pro-eating disorder content. Pro-eating disorder content often considers eating disorders as a lifestyle choice rather than a disorder requiring treatment. Eating disorder–related material can be found on websites and social media platforms such as Facebook, Twitter, and Instagram [15-18], which are now often accessed via apps. Our work differs from prior research in that we focus on an app intended for weight loss instead of technology designed specifically for eating disorders or general-purpose technology (such as social media) that contains eating disorder–related content.

We focus on weight intentions and users’ perceptions of the impact of a weight loss app (DropPounds) on disordered eating behaviors. This study adds to research on weight loss apps and communities, as well as to our understanding of the use of technology by those with disordered eating behaviors and eating disorders.

Two primary research questions (RQ) guided this study, which are as follows:

- RQ1: What is the composition of the overall app community in terms of body mass index (BMI) goals?
- RQ2: What are users with underweight BMI goals perceptions of the app in relation to disordered eating behaviors?

To answer these RQs, we first analyzed users’ profile data to get a better understanding of the composition of the community. Then we provided examples of how users with underweight BMI goals perceive the app. This exploratory study is important for our understanding of the different types of users who utilize weight loss apps and how weight loss apps may impact users with a desire to be underweight. However, more questions emerged, and therefore, future research is needed.

To our knowledge, this study is one of the first to (1) provide an analysis of users’ BMIs in an app and community geared toward weight loss to highlight potential disordered eating behaviors and (2) to consider how users with a desire to be underweight understand weight loss apps in relation to disordered eating behaviors. In this paper, we first explain why we used underweight BMI goals as a marker for potentially disordered eating behaviors. Then we explain our methods and findings. Finally, we discuss what can be learned from this study and future work.

### Desire to Be Underweight and Disordered Eating Behaviors

Using weight loss apps while wanting to be underweight presents a number of issues related to eating disorders and disordered eating behaviors, which is why we focused on underweight BMI goals and the use of weight loss apps to achieve those goals. Whereas anorexia nervosa is partially characterized by being underweight [19] and a refusal to be a healthy weight (as cited in [20]), intentions to be underweight for one’s height itself are problematic. To determine which users wanted to be underweight, we used BMI. For adults 20 years or older, an underweight BMI is less than 18.5 kg/m^2^.

Setting unhealthily low BMI goals signals a desire to be underweight and a drive for thinness, which are associated with disordered eating behaviors and eating disorders [21,22]. Drive for thinness “is characteristic of individuals with fear of weight gain who diet to prevent it, but also of those who seek to attain an unhealthily low body weight as seen in many individuals with anorexia nervosa or bulimia nervosa” [22]. Pro-eating disorder blogs and websites are often characterized by users’ drive to be underweight [23-25]. In terms of goal setting, Boero and Pascoe [21] found that many users of pro-ana (pro-anorexia nervosa) communities write about their maximum, current, and goal weights and are self-identified or diagnosed with eating disorders. The reported goal weights are to be underweight even if users’ current weights are in the healthy or underweight range [21].

The desire to be underweight coupled with the use of weight loss apps may put users at risk for or signal disordered eating behaviors. Weight loss apps encourage dieting behaviors to reach those who need to lose weight. Unfortunately, dieting behaviors are linked to the development of eating disorders [26-28]. In fact, women who severely diet are 18 times more likely to develop an eating disorder, and those who moderately diet are 5 times more likely to develop an eating disorder than those who do not [27]. Studies of adolescent girls have found that high BMI is not a factor for dieting initiation; many girls of a healthy weight and even those who are underweight report wanting to lose weight and to go on a diet [29,30]. Simply wanting to lose weight is associated with disordered eating and weight control behaviors. In her study of US high school students, Forman-Hoffman [31] found that dieting and exercising to lose weight were linked to more unhealthy eating and weight control behaviors [31]. Additionally, over one-third of students who wanted to lose weight also reported one or more of those disordered behaviors [31]. Thus, this drive to be underweight acted as a marker for potential disordered eating behaviors.

## Methods

### Approach and Analysis

We had profile data and forum posts from DropPounds, a mobile- and Web-based weight loss app available on iPhone operating system (OS, Apple Inc.), Android, and through the Web. This app was chosen because at the time the data were provided, it was a popular health app and includes many of the features and content found in the majority of weight loss apps today. For example, DropPounds allows users to track their diet and physical activity. It also has an optional online community associated with it, which users can turn to for advice and support.

We present two pieces of this study that move from a broader picture of the DropPounds community to a narrower view of specific DropPounds users. First, we analyzed users’ profile data to get a sense of the number of users within the community who set underweight goals. From the forum data, we provided examples of users’ posts to highlight their perceptions of the app’s effect on disordered eating behaviors.

Due to the sensitivity around eating disorders, it was important to consider the ethics around conducting research in an online forum with users who may have a history with eating disorders [32]. Both the name of the app and community were changed to protect users’ privacy. Also, we changed the wording of quotations slightly to ensure the anonymity of users while still maintaining the tone and theme of the quotation. As the app company provided the data, we did not have access to users’ information to contact them for their consent.

Institutional review board approval was obtained from 3 universities to conduct the research. The company that owns, maintains, and operates the app gave permission to conduct research and provided app data, including forum posts and portions of users’ profile data. Before providing the data, the company assigned a random unique identifier to users. They did not provide fields that were individually identifying. Forum data and parts of profile data are publicly accessible through the app. Anyone can create an account for free and read posts on the forums. Users input their height, current weight, and then set a goal weight and how many pounds per week they want to lose (up to 2 pounds).

#### Users’ Profile Data

DropPounds provided us with profile data from 19,710 users in 2012. To analyze the overall community, we removed users if they had a BMI under 5 or over 125 (n=14), did not have weight or height data available (n=271), were younger than 20 years (because of the way BMI is calculated for those under 20 years; n=832), or over 99 years (n=1). That left us with 18,601 users. We calculated users’ BMI from the weight and height data in their profile. For adults ≥20 years, underweight is <18.5, healthy weight is 18.5 to 24.9, overweight is 25.0 to 29.9, and obese is 30+. Looking at profile data gave us a better understanding of the composition of the overall DropPounds community.

For the users’ profile data, we used Excel (Microsoft Corp.) to investigate the types and frequency of users’ BMI at the time they created their weight loss plan (start BMI), BMI at the time of data collection (current BMI), and goal BMI.

#### Users’ Posts

We analyzed content from the DropPounds online community for two reasons. First, we wanted to observe whether weight loss app users discussed not only the role of the app but also how it impacted disordered eating behaviors. The examination of forum posts revealed that this phenomenon was occurring. Second, we wanted users’ perceptions without the influence of a researcher asking specific questions. The dataset used in this study comprised 321,999 posts over 24,183 threads that were created from October 2009 to July 2012. Whereas the design of specific features has changed since 2012, the types of features have remained consistent.

To isolate discussions about eating disorders, we used a number of eating disorder–related keywords to identify candidate threads. Keywords included an[eo]rexi[ac], ana, bul[ie]mi[ac], mia, compulsive overeating, body d[iy]smorphi[ac] disorder, bing[e], eating disorder, ED, purg[e], and EDNOS (eating disorder not otherwise specified). The initial keyword search returned 6190 threads representing 9255 unique users. The first author examined the initial set of threads to identify and remove any irrelevant content that the keyword search returned. After removing irrelevant threads, we identified 1036 relevant threads (2678 posts). After removing duplicates (n=342), we had 2336 posts that represented 1080 unique users.

We then pulled every post in the dataset written by users with underweight BMI goals (n=246). After becoming familiar with the data, the first author wrote notes about each post, including the content and disordered eating behaviors mentioned and then created codes based on the content (eg, app impact, binge triggers, and community support). Coded posts were then grouped together. We chose to focus on app impact. We reviewed these posts to see how users discussed the impact of the app on disordered eating behaviors and grouped these posts into two broad categories: (1) reduces disordered eating behaviors and (2) exacerbates disordered eating behaviors. We then broke these up into smaller groups to highlight specific ways the users believe the app reduces or exacerbates disordered eating behaviors, with the purpose of providing example quotations about the app’s impact on disordered eating behaviors for other researchers to use as a basis for future research.

## Results

In this section, we first present statistics about users’ profile data related to their BMI and goals to show the prevalence of users with underweight BMI goals utilizing the app. Then we present examples of how these users discussed the impact of the app on disordered eating behaviors.

### Users’ Profile Data (RQ1)

**Figure 1:**
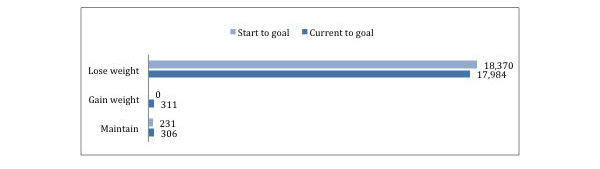
Number of users who had weight loss goals and weight gain goals.

Of 18,601 users, 14,031 identified as female and 4570 as male. The reported age of users ranged from 20 to 99 years (mean=38.42, standard deviation [SD]=11.71, median=37, mode=28). As shown in Figure 1, the majority of users start with weight loss goals (n=18,370), followed by maintenance goals (n=231). None of the users start with weight gain goals. Looking at the change between users’ current BMI to goal BMI, we show most users also have weight loss goals (n=17,984), followed by weight gain goals (n=311) and maintenance goals (n=306). In some instances, users lose more weight than they planned from the time between their program start weight and current weight, so their current BMI to goal BMI reflects a weight gain goal even though their overall objective is to lose weight.

**Figure 2:**
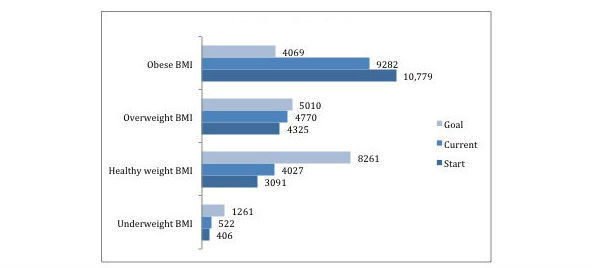
Number of users whose body mass index (BMI) was underweight, healthy weight, overweight, and obese at start, current, and goal.

Within the community, 2.18% (406/18,601) start with underweight BMIs, 2.81% (522/18,601) are currently underweight, and 6.78% (1261/18,601) of the community have a desire to be underweight, which can be seen in Figure 2 along with other information about the start, current, and goal BMIs of the community. The majority of users with underweight BMI goals identify as female (n=1238 compared with n=23 who identify as male). Of those users with underweight BMI goals, most want a BMI of 17 or above (n=585), followed by under 15 (n=289), 16 to 16.99 (n=220), and 15 to 15.99 (n=167).

**Figure 3:**
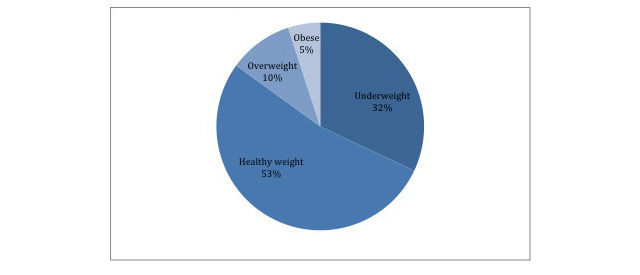
Number of users with underweight body mass index (BMI) goals who have underweight, healthy weight, overweight, and obese start BMIs.

Of the users with underweight BMI goals, the majority (n=671) had healthy start BMIs, followed by underweight start BMIs (n=406), then overweight start BMIs (n=125), and finally obese start BMIs (n=59), as shown in Figure 3. All users with underweight start BMIs (n=406) had underweight goal BMIs; that is, no one who was underweight when they began the program wanted to gain weight to be in the healthy range. Additionally, none of the users with underweight start BMIs had healthy, overweight, or obese current BMIs.

Most users with underweight goal BMIs wanted to lose weight (n=1237), and a small subset wanted to maintain their weight (n=24). None of the users with underweight BMI goals wanted to gain weight. Thus, none were interested in gaining weight even if that allowed them to remain underweight.

### Users’ Posts (RQ2)

We found that 8.98% (97/1080) of users who post in the forums about eating disorders have underweight BMI goals. We then looked at all users with underweight BMI goals and found 7.69% (97/1261) of them post in the forums about eating disorders. Users with underweight BMI goals produced a total of 246 posts (mean=2.54, SD=2.29, range 1-24). Thus, 10.53% (246/2336) of posts about eating disorders were written by users with underweight BMI goals. Seventeen posts from 13 users contained content related to how the app affects disordered eating behaviors. Details on these 13 users are provided in Table 1.

**Table 1:** Users’ age, body mass index (BMI), symptoms, and app perception.

User ID	Reported age, in years	Disordered eating behaviors	Start BMI^a^	Current BMI	Goal BMI	Exacerbates	Reduces
62663	34	Restriction, low weight	12.55	13.11	12.55		X
92214	27	Control food intake	16.09	16.49	14.51	X	
23774	24	Bingeing	17.22	17.22	15.78		X
144279	52	Restriction, bingeing	17.28	15.06	14.62		X
154295	22	EDNOS^b^, restriction, bingeing	17.80	17.47	14.98		X
274788	40	Bingeing	18.16	16.09	15.21		X
533842	40	Anorexia and bulimia nervosa	18.26	15.70	15.21	X	
2042	48	Bingeing	18.46	18.46	16.98		X
29213	20	Restriction, purging	18.75	18.46	16.24	X	
318229	23	Anorexia and bulimia nervosa	19.57	18.47	18.23	X	
172884	41	Former bulimia nervosa	20.90	20.01	18.07	X	X
215596	54	Bingeing, emotional eating	21.43	18.04	18.04		X
397752	24	Bingeing	22.78	20.88	18.31		X

^a^BMI: body mass index.

^b^EDNOS: eating disorder not otherwise specified.

All users with underweight BMI goals who posted about the effects of the app in the forum identified as female. The majority of these users began the program underweight (n=8), followed by healthy weight (n=5). For current BMI, most users fell into the underweight category (n=11), followed by healthy weight (n=2). Eight users discussed how the app helps reduce disordered eating behaviors, 4 users discussed how the app exacerbates disordered eating behaviors, and 1 user talked about how the app did both. We provide example posts that highlight these perceptions of the users about the app in Tables 2 and 3.

More often than not, users felt that DropPounds was a positive influence because it created awareness and accountability that reduced bingeing, helped them eat more, improved their food choices, and provided them with a healthy plan. Many posts focus on bingeing behaviors, so many users felt the app helped them control those behaviors and choose healthier foods overall. Users with a history of extreme calorie and food restriction felt that the app gave them awareness about their restrictive behaviors, which helped them see where they should add foods. Some users believed the daily calorie budget was inherently healthy. Table 2 provides example posts from users who discussed these positive aspects of the app.

**Table 2:** Positive aspects of the app and example posts.

Positive aspects	Example post
Reduces bingeing	“The best thing for me for emotional eating or binge eating is logging! If I am faithfully logging, I have much better control over that stuff because I don’t want to enter a bunch of crap. I am really proud of myself for not succumbing to those desires to binge.” [ID 215596]
Helps eat more	“I suffered from disordered eating my entire life. My eating issues were never extreme enough to be considered full-blown eating disorders but were enough to have a big negative effect on my life. Until I started using DropPounds, it was almost impossible for me to eat 3 meals a day. My usual pattern included starving myself and then bingeing, compulsive eating, and sporadic, unsustainable diets. My self-esteem has always been tied to my weight and whether I had a ‘good eating day’ or a ‘bad eating day.’ Since I have been on DropPounds, I have finally learned how to eat 3 meals a day (and snacks). Every meal and mouthful is still a battle but at least I’m finally winning the fight.” [ID 144279]
Improves food choices	“DropPounds for me is more about being held accountable for my food choices, as I have a bit of a sugar issue and tendency to binge until I feel ill. This is about making sure I get enough fruits, vegetables, fiber **,** and avoid eating mindlessly.” [ID 23774]
Provides a healthy plan	“I have EDNOS, and I’m trying to recover. This app and community really motivate me to lose weight in a healthy manner. Unfortunately, I purged last night, but today I didn’t. At one point, I used to purge everything I ate no matter what it was: fruit, vegetables, diet coke, and water. I’m motivated and inspired to finish recovery by myself because I had a negative experience in a hospital. I’m very happy here, and I love how it [DropPounds] gives you the amount of calories to eat. You can still lose weight, and it selects a goal for you, which makes it healthy.” [ID 154295]

Some users also discussed how the app could exacerbate disordered eating behaviors. For example, the app encourages purging calories through excessive exercise by providing negative feedback when users exceed their budget and allowing them to erase calories to receive positive feedback. Not only does it promote compensatory behaviors, but it also encourages users to eat less than their allotted budget. Having disordered eating behaviors in combination with using the app also could lead to or exacerbate obsessive behaviors and thoughts around logging and numbers. Whereas some users felt the app’s algorithm automatically meant that the plan was healthy, other users suggested that the app’s goal-based plan was actually unhealthy. Table 3 provides example posts of these negative aspects. These findings provide a preliminary look into how apps may reduce and exacerbate disordered eating behaviors.

**Table 3:** Negative aspects of the app and example posts.

Negative aspects	Example post
Encourages purging	“The times where my bar showed I was over calories, I would punish myself with an extensive amount of exercise while talking down to myself. There was a times where I would go over an insignificant amount of calories, 50 perhaps, and punish myself with a large amount of unnecessary exercising.” [ID 29213]
Promotes eating less	“I have found myself doing this [trying to eat less and less like it’s a game] and have to remind myself daily (usually at every meal) that it’s not about the number; it’s about making healthy choices **.** I have to make myself not feel like an utter failure if I don’t stay under my calorie limit.” [ID 533842]
Leads to obsessive behaviors	“I have struggled with bulimia/anorexia for the past 4 to 5 years, and I still struggle today in being happy with my body. I am a perfectionist and have a somewhat obsessive personality so I can get obsessed with logging my food and thinking about how many calories I am eating and drinking at all times. I probably shouldn’t be on this site sometimes!” [ID 318229]
Provides a dangerous plan	“Many people who frequent these forums know about the 1200/1500 calorie minimum for women and men, but those who have not joined the forums only assume that the less they eat, the more they will lose. For example, when I began this program over a year ago, I set my goal to 2 pounds per week in order to get things accomplished faster. My budget was around 900 calories, which I ate. DropPounds is the one calculating the calories people consume. While we cannot solely blame DropPounds for its cold calculation, we have to consider the ignorance of many people who are using this program and who are destroying their well-being in the process.” [ID 29213]

## Discussion

### Principal Findings

In summary, we found three main results: (1) no user (regardless of start BMI) starts with a weight gain goal, and most users want to lose weight; (2) 6.78% (1261/18,601) of the community want to be underweight, and most identify as female; (3) users with underweight BMI goals tend to view the app as positive; however, some acknowledge its role in exacerbating disordered eating behaviors. In this section, we discuss these findings and present a number of areas that researchers and designers need to consider in more detail.

### Weight Loss and Underweight Goals

We found that no user set a weight gain goal. In fact, the vast majority of the community (98.76%; 18,370/18,601) begins the program to lose weight (not maintain or gain). The users who are underweight when they begin the program do not want to gain weight, and most of these users want to lose additional weight, which would put them at a more extreme low weight for their height. A small subset of users with underweight BMI goals is using the app to maintain an already low weight. No one using the app is doing so to gain weight even if they should gain weight or report needing to gain weight as part of their eating disorder recovery. Thus, the overall focus of app use is weight loss irrespective of the start weight of users.

In addition to the heavy focus of weight loss by all users, we found that 6.78% (1261/18,601) of the users wish to be underweight according to BMI. The majority of users who set underweight goals begin the program at either a healthy weight or are already underweight, and most of these users identify as female. These findings suggest women often want to lose weight even when weight loss is unnecessary, which is in line with prior research [31,33]. This may be explained in part by women’s body dissatisfaction and weight perception. It is common for women to be discontent with their bodies and weight. In fact, in North America, there is such a pervasive body dissatisfaction and preoccupation with weight among women that psychologists have developed a term “normative discontent,” which describes the normalcy of being unhappy with one’s weight as a woman [34].

Research has also shown that women and girls tend to have an inaccurate perception of their weight; they perceive their weight as higher than it actually is [30,31,35]. For example, in their study of first-year college women, Cilliers et al [30] found that only a few in the healthy weight range were satisfied with their weight, and many wanted to lose weight, which could result in their engaging in unnecessary and unhealthy weight control practices. A quarter of the underweight students also still wanted to lose weight, and the remaining underweight students wanted to keep their underweight status [30]. Similarly, Forman-Hoffman [31] found that over 20% of high school students overestimate or extremely overestimate their weight. When women and girls perceive their weight as higher than it is, they may wish to lose weight that would put them at an unhealthy weight for their height. This overestimation is linked to disordered eating behaviors [31]. Thus, users’ goals may explain a lot about them. To promote health, researchers should further examine the relationship between perceived weight, goal weight, and eating disorders to find ways to empower users to make healthy choices and set healthy goals.

Although DropPounds and other weight loss apps and communities are designed for users who need to lose weight, our study shows that users who likely do not need to lose weight but are dissatisfied with their current weight are using the app to achieve unhealthily low weights. This is an important finding because designers and developers often focus on the intended and “ideal” user, which makes sense, given the focus of the app (weight loss); however, our study shows that we need to pay more attention to unintended or “nonideal” users and the uses, perceptions, and effects of weight loss apps on them.

### App Perceptions

We provided example posts from users who discussed the app as positive and negative. These examples are not meant to be an exhaustive list of the use and perceptions of weight loss apps; instead the intention is that these provide a basis for researchers to thoroughly investigate the role of weight loss apps for those with disordered eating behaviors. Researchers can use these eight themes (reduces bingeing, helps eat more, improves food choices, provides a healthy plan, encourages purging, promotes eating less, leads to obsessive behaviors, and provides a dangerous plan) to examine aspects and features of weight loss apps that could be useful for eating disorder recovery or aggravate disordered eating behaviors.

Similar to Tan et al [15], we found that users discussed the weight loss app as reducing disordered eating behaviors and exacerbating them; however, more users talked about the positive aspects of the app than the negative aspects. These findings are both in line with and in opposition to interview-based research about the use and perceptions of weight loss apps by women with eating disorders. Interview-based findings support the idea that weight loss apps encourage purging or compensatory behaviors, promote restriction, and lead to obsessive logging [14]. However, this study did not discuss dangerously low plans, as the focus was on a different app that did not allow users to set a daily calorie budget below 1200 [14], suggesting that having minimum daily calorie budgets may be beneficial to users, especially those who are unaware of how many calories they need. Additionally, positive aspects of the app included its ability to show users how much they needed to eat, much like this study; however, the set healthy plan did not emerge as a major finding [14].

So why do users in this study tend to view the app as mostly positive? One possible explanation may be related to the type of disordered eating behaviors. Of the 13 users, 10 mentioned bingeing or bulimia-related behaviors. The app may, in fact, be beneficial to promote mindfulness, and logging foods during a binge may reduce the amount of food users eat. However, given that these users have a desire to be underweight, the app may contribute to or exacerbate other behaviors. Thus, more research is needed to differentiate the effects and uses related to types of disordered eating behaviors and eating disorders. For instance, creating awareness may be beneficial for those with binge eating disorder, but this awareness could be problematic for those who have a high drive for thinness, fear of weight gain, anorexia nervosa, and so on.

Another possible explanation may relate to users’ current stage of their disordered eating or eating disorder and their ability to reflect on their behaviors. When users post in the forums about the app, they may feel as though the app is helpful to them even if their behaviors are disordered. They may be at a stage where they do not recognize their disordered eating behaviors or are unwilling to. This is echoed in the concept of a user’s health journey [14]. In the interview-based work, participants were reflecting on their use over time, and they often explained that at the beginning of app use they saw no problem with their behaviors or the app but later realized the app was exacerbating their disordered behaviors more than helping them [14]. Thus, it is possible that the users in this study were in the earlier stages of use when they discussed the app in the forums. More research is needed to understand the effects of weight loss apps on disordered eating behaviors and how the stages of the users’ health journey change the role technology plays.

### Discrepancies Between App Perceptions and Goals

On the basis of this study alone, we do not understand why users set low weight goals. Some users explicitly stated needing to gain weight for recovery but had weight *loss* goals. For example, users with underweight start BMIs and underweight goal BMIs said that the technology is helping to reduce their eating disorder behaviors, and every user with an underweight start weight had an underweight goal weight. This begs the question: do some users really want to recover? We cannot determine whether or not users truly want to use the app to reduce their disordered eating behaviors. Our results indicate that women’s discussions of eating behaviors in the DropPounds forum often focused on being healthy, and no user in the sample openly said they were trying to maintain disordered eating behaviors. However, their private weight goals would put them at an underweight BMI.

The tension between users’ private goals and forum posts may be related to self-presentation. According to Counts and Stecher [36], “self-presentation can be thought of as the image or idea of the self, or the process of creating this image for a variety of social purposes.” Research has shown that people present themselves in desirable ways when they are in the public [37]. Self-presentation motives include achieving your goals, presenting a positive view of self to the world, and conforming to social norms. According to Goffman [37], life is comparative to the theater: people do “front stage” work when they are interacting with others in public settings, and they do “back stage” work, which comprises the private things people do when no one is looking. These two presentations can be misaligned. In this case, the forum posts represent “front stage” work, whereas the private profile goals represent “back stage” work.

Although research has shown that eating lightly to achieve thinness is desirable for women [38], having an eating disorder or disordered eating behaviors in a community whose focus is health may be undesirable. This is in line with prior work on self-presentation in an online community. Schwammlein and Wodzicki [39] found that “members of the common-identity community focused on characteristics shared among members of the community” versus those who were part of the common-bond community. The DropPounds community shares a common identity: its users emphasize *healthy* weight loss. Many of its users are serious about maintaining a healthy lifestyle and are against unhealthy or extreme tactics to lose weight. Due to this, users who may have disordered eating behaviors and want to post in the forums may attempt to conform to the community’s norms and its members’ opinions and characteristics. Many more may choose not to post in the forums about their disordered eating behaviors because of fear of backlash from the rest of the community or privacy concerns. Research has shown that women with eating disorders may be reluctant to use social and community features of weight loss apps [40]. This may explain why only 97 users with underweight BMI goals were present in the forums even though 1261 users set underweight BMI goals and why only 13 of these users talked about the impact of the app within the forums.

We cannot determine whether users are actively trying to maintain their disordered eating behaviors or whether they want to recover but are not setting appropriate goals. There may be a subset of users who do not want to recover from their eating disorder or disordered eating behaviors. Weight loss apps and forums can give them a false feeling that their behaviors are healthy, which can allow them to deny they have disordered eating behaviors. Researchers need to investigate in more detail why users with disordered eating behaviors use weight loss apps and their intentions regarding eating disorder maintenance and recovery.

Another potential explanation comes out of the interview-based study [14]. In that study, users discussed trying to use the app for recovery but falling back into old habits [14]. Therefore, some users may in fact be trying to recover while combatting the urge to lose weight and restrict calories. This may, in part, be because of the nature of weight loss apps. Through design, users are supposed to be motivated to lose weight, so even when they set weight gain goals or attempt to eat more calories, the app still shows visualizations to motivate them to lose weight. Thus, more research is needed on the effects of design and how we motivate users through design. Health app designers should also consider ways to promote other types of goals besides weight loss. Weight loss is not the only type of health and fitness goal. For instance, users may wish to gain muscle or use the app to achieve exercise-related goals not even tied to a goal weight.

### Limitations

There are a few limitations of this research, including sample size, date of data collection, and using BMI. Although there were over 1200 users who had underweight BMI goals, not every user posted in the forums. Analyzing the profile and forum data meant that we were unable to get the perspectives of users with disordered eating behaviors who did not post in the forum. Additionally, for the purposes of this study, we focused on users’ discussions on the impact of the app. Thus, we could only provide example quotations from a small subset of users. This likely does not cover all perceptions of the app but is meant as a jumping point for future research.

Another limitation is the age of the data. Whereas certain features of the app have changed since the time of data collection, the overall focus of the app is the same. The app still contains a food, exercise, and weight loss log and shows progress visualizations based on these factors, but the look of the app has changed. However, the focus of this research was not on specific design features or aesthetics, so the findings are still relevant not only to DropPounds but also to other weight loss apps. Since data collection, the app’s popularity has skyrocketed. Thus, we suspect even more users with disordered eating behaviors are utilizing the app.

Although BMI has its limitations, BMI is used in the Diagnostic and Statistical Manual of Mental Disorders, 5th edition (DSM-V) to aid in the diagnosis of eating disorders [19]. Additionally, per the Centers for Disease Control and Prevention (CDC), BMI below 18.5 is considered underweight [41]. We chose to focus on users with underweight BMI goals because this desire to be underweight can signal unhealthy behaviors. Having an underweight BMI goal does not mean that all of these users have clinical eating disorders; rather the intent to be underweight and wanting to lose weight when already at a healthy weight or underweight could be indicative of disordered eating behaviors [21,22]. Additionally, BMI under 18.5 is a potential marker of anorexia nervosa. Thus, we used goal BMI as an indicator of disordered eating behaviors. However, it is likely that we missed users with disordered eating behaviors who have goal weights that put their BMI in the healthy, overweight, or obese range, which means that there are potentially many more users with disordered eating behaviors using weight loss apps. The aim of this study was to focus on weight intentions, specifically users with underweight BMI goals, and shed light on their app perceptions. Despite the limitations, this study is a good first step toward looking at the composition of weight loss app communities and the impact of weight loss apps on disordered eating behaviors.

### Future Work

This research represents one phase of a larger project. We are also conducting interviews with weight loss app users with eating disorders. From the profile data, we found that many users had either underweight BMI start weights or underweight BMI goals, which suggests a large prevalence of users with disordered eating behaviors. However, most of these users did not post about eating disorders in the forums. Thus, conducting interviews allows us to get a larger sample size as well as ask additional questions about how users utilize weight loss apps and how those apps impact them. After we conduct the interviews, we plan to get a broader view of the phenomenon by conducting a survey, which will help with generalizability.

### Conclusions

In this study, we looked at the underweight BMI goals of users in a weight loss community and perceptions of these users with regard to the impact of a weight loss app on disordered eating behaviors. A number of users within the community had underweight BMI goals, suggesting they have a strong drive for thinness and a desire to be underweight, which may signal disordered eating behaviors. Users with underweight BMI goals tend to view the app as beneficial for disordered eating behaviors, especially bingeing. Although users with underweight BMI goals felt different features of the app could both reduce and exacerbate disordered eating behaviors, their overall perceptions and goals were misaligned. While this study provides examples of how weight loss apps may impact disordered eating behaviors, a number of questions emerged leading to suggestions for additional research directions. As this is an understudied area, more work is needed on the use of weight loss apps by users with eating disorders and disordered eating behaviors. Our future work aims to explore this area more thoroughly. We hope that this study sparks more research on the role of technology for users with disordered eating behaviors.

## Abbreviations

BMI: body mass index
CDC: Centers for Disease Control and Prevention
DSM-V: Diagnostic and Statistical Manual of Mental Disorders, 5th edition
EDNOS: eating disorder not otherwise specified
mHealth: mobile health
OS: operating system
RQ: research question
SD: standard deviation
